# Effect of harvest time on sugar content and carotenoid composition in different sweet maize hybrids

**DOI:** 10.1038/s41598-025-11680-w

**Published:** 2025-07-16

**Authors:** Seyed Mohammad Nasir Mousavi, Arpad Illes, Csaba Bojtor, Younes Miar, Seyed Habib Shojaie, Janos Nagy, Adrienn Szeles

**Affiliations:** 1 Institute of Land Use, Engineering and Precision Farming Technology, University of Debrecen, 138 Böszörményi St, Debrecen, 4032 Hungary; 2 Department of Animal Science and Aquaculture, Dalhousie University, B2N 5E3 Truro, NS Iran; 3 Department of Biotechnology and Plant Breeding, Faculty of Agriculture and Food Science and Technology, Science and Research Branch, Islamic Azad University, Tehran, 1477893855 Iran

**Keywords:** Zeaxanthin, Carotenoid, Nutrient, Sweet maize, Plant sciences, Plant breeding

## Abstract

Sweet maize (Zea mays convar. saccharata var. rugosa) is valued for its high sugar content, which gradually converts to starch during kernel maturation. This study evaluated the yield and biochemical composition of five super sweet maize hybrids (D, M, G, S, and N) across four harvest dates (July 19, July 26, August 2, and August 9) over two consecutive growing seasons. Seventeen key nutritional parameters—including sugars, minerals, and carotenoids—were significantly affected by both hybrid and harvest time (*p* < 0.05). The M hybrid showed the highest levels of β-carotene (5.61 µg/g), phosphorus (3.45 mg/g), and magnesium (1.96 mg/g), while the S hybrid had the greatest concentrations of lutein (2.84 µg/g), zeaxanthin (2.71 µg/g), and β-cryptoxanthin (1.79 µg/g). The D hybrid recorded the highest sucrose content (78.5 mg/g), and the N hybrid was superior in dry matter (32.7%) and fructose (61.4 mg/g). Harvest timing also had a significant impact: early harvest (July 19) resulted in peak concentrations of β-carotene (5.89 µg/g), potassium (4.17 mg/g), and glucose (84.2 mg/g), whereas late harvest (August 9) favored hybrid-specific nutrient accumulation. Principal Component Analysis (PCA) revealed that the first two components explained 75.82% of the total variance, with glucose and potassium identified as key discriminating traits. These findings highlight the critical role of genotype selection and harvest timing in optimizing the nutritional quality and market value of sweet maize under temperate growing conditions.

**Supplementary Information:**

The online version contains supplementary material available at 10.1038/s41598-025-11680-w.

## Introduction

Sweet maize (Zea mays convar. saccharata), commonly referred to as sweet corn, is harvested at the immature or milk stage, when the kernels are soft, tender, and rich in natural sugars. Unlike field corn, which is grown primarily for dry grain, silage, or industrial purposes, sweet maize is cultivated for direct human consumption as a fresh vegetable. Its pleasant flavor, texture, and ease of preparation make it a dietary staple in many regions worldwide. Beyond its sensory appeal, sweet maize offers nutritional benefits, providing dietary fiber and essential minerals such as potassium and magnesium—nutrients important for cardiovascular health, neuromuscular function, and bone metabolism^1,2^. Due to its adaptability to diverse climates, sweet maize is widely cultivated in both temperate and tropical zones, where it supports local food systems, generates seasonal employment, and contributes to food security and export markets^3^. In recent years, increasing attention has focused on the functional properties of sweet maize, particularly its content of bioactive compounds such as carotenoids. These plant pigments, responsible for the yellow to orange coloration in fruits and vegetables, are broadly categorized into two groups: provitamin A carotenoids (e.g., β-carotene, α-carotene, and β-cryptoxanthin), which can be metabolized into vitamin A, and non-provitamin A carotenoids (e.g., lutein and zeaxanthin), which play other key physiological roles^4–6^. Carotenoid intake has been linked to enhanced immune response, reduced inflammation, and protection against oxidative stress and chronic diseases. Notably, lutein and zeaxanthin accumulate in the human retina, where they filter harmful blue light, reduce photooxidative damage, and help prevent age-related macular degeneration (AMD)—a leading cause of vision loss in older adults^7–10^. Although sweet maize is not the most carotenoid-rich vegetable compared to leafy greens or orange-fleshed crops, its wide consumption and cultural acceptance make it a practical dietary source. Moreover, recent advances in plant breeding and biofortification have yielded sweet maize hybrids with significantly elevated carotenoid content, particularly β-carotene and zeaxanthin. These biofortified varieties retain desirable agronomic and sensory traits while enhancing micronutrient intake in populations at risk of vitamin A deficiency^11,12^. Despite these advances, several knowledge gaps persist. One critical yet understudied factor influencing sweet maize nutritional quality is harvest timing. The stage at which sweet maize is harvested directly affects its sensory characteristics and biochemical composition. In early kernel development, sugars such as sucrose, glucose, and fructose accumulate rapidly, contributing to sweetness. As the kernels mature, these sugars are gradually converted to starch, reducing sweetness and increasing firmness. Likewise, carotenoid levels fluctuate throughout development, although the patterns of accumulation and degradation are not fully understood. Optimizing harvest timing is therefore essential to balance consumer preferences with nutritional quality. This study aims to address these gaps by systematically evaluating how harvest timing affects the yield, sugar content, and carotenoid composition of five super sweet maize hybrids. Specifically, the objectives are to:

1. Quantify changes in sugar and carotenoid profiles across multiple harvest intervals, and.
2. Assess hybrid-specific responses to harvest timing, identifying genotypes with superior nutritional stability across maturity stages.

We hypothesize that harvest timing significantly influences sweet maize’s nutritional composition and that some hybrids retain higher levels of sugars and carotenoids across developmental stages. The findings aim to support breeders, producers, and stakeholders in optimizing sweet maize production for both nutritional value and market appeal, contributing to broader efforts in improving staple vegetable crops through targeted agronomic practices and varietal selection.

## Materials and methods

### Experimental site and soil characterization

The field experiment was conducted over two consecutive growing seasons (April–August 2022 and 2023) at the Látóképi Research Farm, Faculty of Agriculture, Food Science and Environmental Management (DE MÉK), University of Debrecen, Hungary (47°33′ N, 21°27′ E; 114 m a.s.l.). The soil at the site is classified as calcareous Chernozem, developed from loess parent material. Prior to planting in each season, composite soil samples (0–30 cm depth) were collected and analyzed at the DE MÉK Accredited Soil Laboratory. The key soil properties (mean ± standard deviation) were as follows: pH (KCl) 6.7 ± 0.1, organic matter content 3.2 ± 0.2%, total nitrogen 0.19 ± 0.01%, available phosphorus (Olsen) 22 ± 3 mg kg^−1^, available potassium (AL) 240 ± 15 mg kg^−1^, calcium carbonate (CaCO₃) content 3.4 ± 0.4%, and cation-exchange capacity (CEC) 28 ± 2 cmol ± kg^−1^. The soil texture was classified as silty loam, comprising 18% clay, 58% silt, and 24% sand (Table 1). The sweet maize hybrids used in the study were obtained from the Precision Agriculture Institute at the University of Debrecen.

**Table 1:** Selected physicochemical properties of the experimental soil.

Parameter	2022	2023	Method
pH (KCl)	6.8	6.7	MSZ-08-0206
Organic matter (%)	3.3	3.1	Walkley–Black
Total N (%)	0.19	0.18	Kjeldahl
Available P (mg kg^−1^)	23	21	Olsen
Available K (mg kg^−1^)	246	234	Ammonium-lactate

**Fig. 1:**
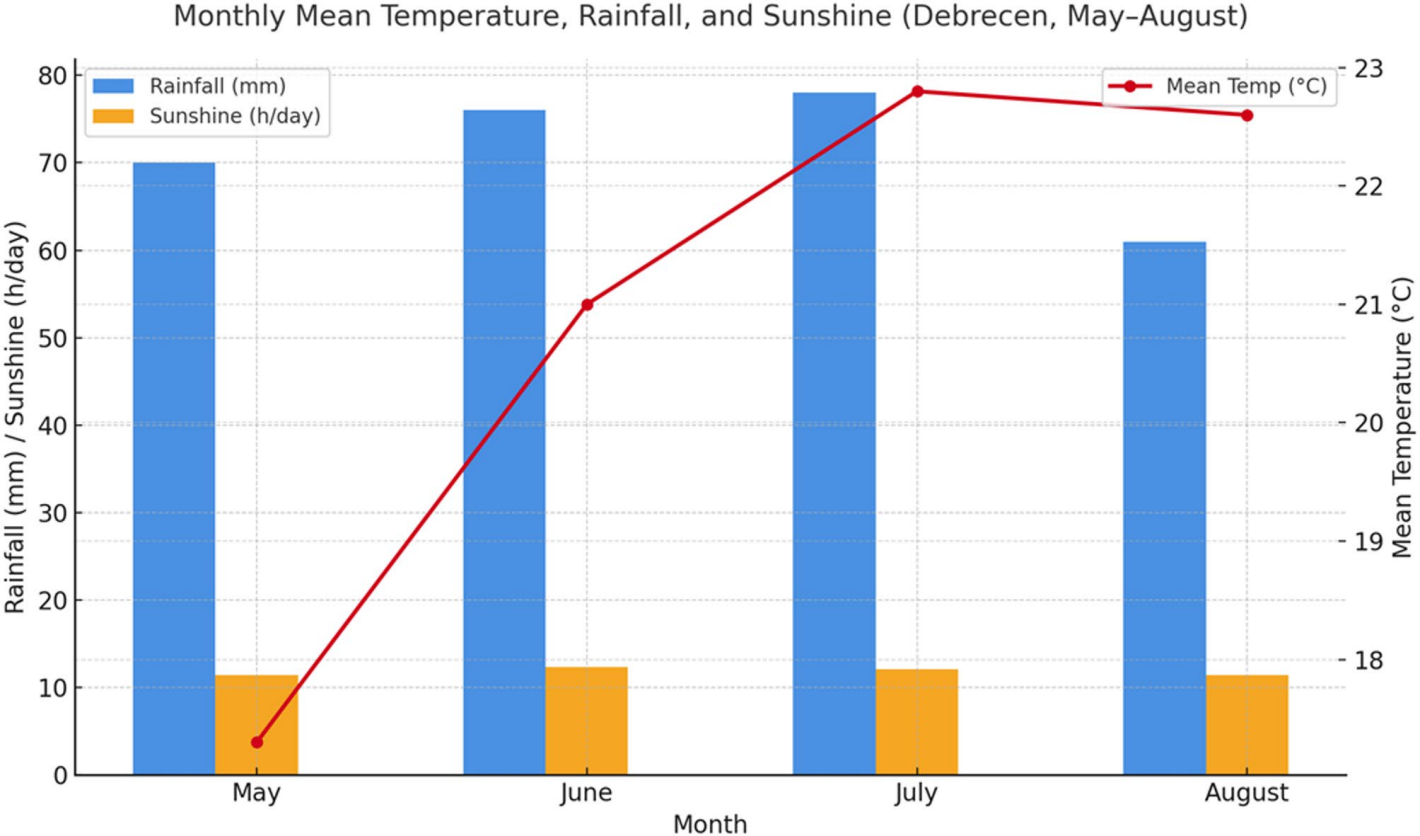
Monthly mean temperature, rainfall and solar radiation during the two seasons.

Daily weather data were recorded using a HOBO RX3000 meteorological station located less than 200 m from the experimental site. Compared to the 30-year climatic average for Debrecen, the 2022 growing season was slightly warmer and drier, while 2023 was markedly warmer with near-average precipitation (Fig. 1). During the main growing period (May–August), the mean air temperature was 25.6 °C in 2022 and 28.3 °C in 2023. Cumulative rainfall, excluding irrigation, totaled 146 mm in 2022 and 157 mm in 2023. Global solar radiation during the same period reached 2,035 and 2,112 MJ m^−2^, respectively.

### Experimental design and crop management

The experiment was laid out in a randomized complete block design (RCBD) with four replications. Each plot comprised four rows, each 6 m long and spaced 0.70 m apart, resulting in a total plot area of 16.8 m^2^. Seeds were precision-sown at a within-row spacing of 24 cm, corresponding to a plant density of approximately 60,000 plants ha^−1^. To minimize border effects, the central two rows (4.2 m^2^) were designated as the harvest area, while the outer rows and 0.8 m-wide alleys between plots served as buffer zones. Five commercially relevant super sweet maize (sh2) hybrids—designated as ‘D’, ‘M’, ‘G’, ‘S’, and ‘N’—were used as experimental treatments. All agronomic practices, including fertilization, irrigation, and pest management, were uniformly applied across treatments to ensure comparability.

### Fertiliser programme

A basal fertiliser application was broadcast and incorporated into the soil one week prior to sowing. This included 60 kg ha^−1^ nitrogen (N), 60 kg ha^−1^ phosphorus (P_2_O_5_), and 80 kg ha^−1^ potassium (K_2_O), applied as urea, triple superphosphate, and potassium sulphate, respectively. An additional 60 kg ha⁻¹ of nitrogen was side-dressed at the V6 growth stage (approximately 30 days after emergence) using a urea–ammonium nitrate (UAN) solution. To prevent micronutrient deficiencies commonly observed in calcareous Chernozem soils, foliar applications of zinc sulfate heptahydrate (ZnSO_4_·7 H_2_O, supplying 10 kg ha^−1^ Zn) and boric acid (2 kg ha^−1^ B) were carried out at the V8 stage. All fertiliser rates were based on local agronomic recommendations for sweet maize and were applied uniformly across all hybrids and both growing seasons.

### Irrigation and other cultural practices

Soil moisture in the 0–40 cm root zone was monitored using capacitance sensors (Decagon 5TM) installed in representative plots. Irrigation was triggered automatically via the Hydrawise^®^ remote-control system whenever volumetric water content declined below 70% of field capacity (approximately 22% v/v). Total supplemental irrigation applied amounted to 283.8 mm in 2022 and 298.2 mm in 2023. Weed control was managed with a pre-emergence herbicide mixture of S-metolachlor (1.6 kg a.i. ha^−1^) and terbuthylazine (0.5 kg a.i. ha^−1^), followed by manual hoeing as necessary. No significant pest or disease outbreaks were observed during either growing season, and thus no crop protection sprays were required.

### Harvest scheduling and sampling strategy

To evaluate the effect of maturity stage on sweet maize biochemical composition, four harvest dates were selected corresponding to key kernel developmental stages: kernel milk (A – 19 July), early dough (B – 26 July), full dough (C – 2 August), and early dent (D – 9 August), based on the phonological scale described by Gironde et al.^13^. At each harvest date, 10 marketable cobs per plot were randomly harvested from the two central rows, avoiding border plants to reduce edge effects. The harvested cobs were immediately transported on ice to the laboratory to preserve sample integrity. Thus, for each hybrid per season, a total of 160 cobs were collected (10 cobs × 4 harvest dates × 4 replications). For chemical analyses, kernels from every two cobs were pooled to form five biological replicates, and each biological replicate was analyzed in triplicate (technical replicates) for HPLC-based sugar profiling, ICP-AES mineral content, and carotenoid composition.

### Post-harvest sample preparation

After harvest, cobs were husked and kernels were manually removed. The kernels were immediately frozen in liquid nitrogen (N₂) and subsequently lyophilized using a Labconco FreeZone freeze dryer (USA). The dried samples were milled to a particle size of less than 0.5 mm using a Retsch ZM200 grinder and stored at − 80 °C in the dark until further analysis. Moisture content was determined according to the AOAC Official Method 934.01.

### Chemical analyses

#### Mineral elements

Exactly 0.50 g of flour was digested with 5 mL of 30% hydrogen peroxide (H_2_O_2_) and 5 mL of concentrated nitric acid (HNO_2_) in a microwave digestion system (ETHOS Plus, Milestone) following Application Note 076. The resulting digest was diluted to 50 mL with deionized water and analyzed by inductively coupled plasma atomic emission spectroscopy (ICP-AES) using an iCAP 7400 instrument (Thermo Fisher Scientific). The emission wavelengths monitored were: Ca (317.933 nm), Fe (238.204 nm), K (769.896 nm), Li (670.784 nm), Mg (285.213 nm), Na (589.592 nm), P (177.495 nm), and Zn (213.856 nm). Quality control was ensured by analyzing the certified reference material NIST 1515 (apple leaves), with recoveries ranging between 95 and 104%.

#### Soluble sugars

Fructose, glucose, and sucrose were quantified using high-performance liquid chromatography with refractive index detection (HPLC-RI; Agilent 1200). Samples were clarified by Carrez I and II precipitation and filtered through 0.22 μm membranes prior to analysis. Separation was performed on an Aminex HPX-87P column (Bio-Rad) maintained at 80 °C, with ultrapure water as the mobile phase at a flow rate of 0.6 mL min^−1^.

#### Carotenoids

Lutein, zeaxanthin, and β-cryptoxanthin were extracted under dark conditions using a hexane/ethanol/NaCl solvent system as described by Kurilich and Juvik (1999), with 0.1% butylated hydroxytoluene (BHT) added to prevent oxidation. Carotenoid quantification was carried out by HPLC with diode-array detection (HPLC-DAD) using a Waters Alliance 2695 system fitted with a YMC C30 column (250 × 4.6 mm, 5 μm particle size). External standards (Sigma-Aldrich) were used for calibration.

### Statistical analysis

Separate and combined analyses were performed across the two growing seasons. Two-way ANOVA was used to evaluate the effects of hybrid (H) and harvest date (D), while three-way ANOVA (H × D × Year) assessed the stability of responses across years. Assumptions of homogeneity of variance and normality were tested using Levene’s test and the Shapiro–Wilk test, respectively. Percentage data were arcsine-transformed as needed to meet ANOVA assumptions. Mean comparisons were conducted using Tukey’s Honest Significant Difference (HSD) test at a significance level of *p* ≤ 0.05 with GenStat 22 (VSN International). Pearson correlation matrices among yield, sugars, carotenoids, and mineral traits were calculated using XLSTAT 2024. Principal component analysis (PCA) was performed in GenStat to visualize multivariate relationships, with sampling adequacy confirmed by Kaiser-Meyer-Olkin (KMO) values > 0.70 and components retained based on eigenvalues > 1. The first three principal components accounted for ≥ 80% of the total variance.

## Results

### Variance analysis and grouping LSD

Variance analysis showed that dry matter, fructose, glucose, sucrose, calcium, iron, potassium, magnesium, zinc, phosphorus, lutein, zeaxanthin, β-cryptoxanthin, α-carotene, 9Z-β-carotene, and β-carotene were significantly influenced by both hybrid and sampling time (Table 2). However, the interaction between hybrid and sampling time was not significant for β-carotene and 9Z-β-carotene, whereas it was significant for all other traits.

LSD grouping revealed distinct differences among hybrids: Hybrid M exhibited superior accumulation of β-carotene, 9Z-β-carotene, α-carotene, phosphorus, zinc, magnesium, potassium, iron, calcium, and glucose. Hybrid S had the highest concentrations of β-cryptoxanthin, zeaxanthin, and lutein. Hybrid D showed the greatest sucrose content. Hybrid N contained the highest fructose and dry matter levels.

Regarding sampling times: Sampling time A (kernel milk) resulted in the highest accumulation of β-carotene, 9Z-β-carotene, α-carotene, phosphorus, zinc, magnesium, potassium, calcium, glucose, and fructose. Sampling time B showed the highest iron content. Sampling time C had the maximum lutein concentration. Sampling time D (early dent) exhibited peak β-cryptoxanthin, zeaxanthin, sucrose, and dry matter (Appendix A). These findings emphasize the significant roles of both genotype and harvest timing in determining the nutritional and biochemical composition of sweet maize).

**Table 2:** Variance analysis among biochemical and nutritional parameters in crop Samples.

	Source	DF	F-Value	*P*-Value
Dry matter	Hybrids	4	308.48^**^	*p* < 0.01
	sampling time	3	3479.96^**^	*p* < 0.01
	Hybrids*sampling time	12	25.42^**^	*p* < 0.01
Fructose	Hybrids	4	319.45^**^	*p* < 0.01
	sampling time	3	1854.98^**^	*p* < 0.01
	Hybrids *sampling time	12	44.58^**^	*p* < 0.01
Glucose	Hybrids	4	377.38^**^	*p* < 0.01
	sampling time	3	2086.28^**^	*p* < 0.01
	Hybrids *sampling time	12	60.46^**^	*p* < 0.01
Sucrose	Hybrids	4	402.97^**^	*p* < 0.01
	sampling time	3	1281.53^**^	*p* < 0.01
	Hybrids *sampling time	12	46.16^**^	*p* < 0.01
Calcium	Hybrids	4	3359.65^**^	*p* < 0.01
	sampling time	3	49400.71^**^	*p* < 0.01
	Hybrids *sampling time	12	366.35^**^	*p* < 0.01
Iron	Hybrids	4	328.94^**^	*p* < 0.01
	sampling time	3	44.22^**^	*p* < 0.01
	Hybrids *sampling time	12	139.17^**^	*p* < 0.01
Potassium	Hybrids	4	270.06^**^	*p* < 0.01
	sampling time	3	883.37^**^	*p* < 0.01
	Hybrids *sampling time	12	39.13^**^	*p* < 0.01
Magnesium	Hybrids	4	2349.06^**^	*p* < 0.01
	sampling time	3	2115.86^**^	*p* < 0.01
	Hybrids *sampling time	12	168.65^**^	*p* < 0.01
Zinc	Hybrids	4	682.20^**^	*p* < 0.01
	sampling time	3	1151.78^**^	*p* < 0.01
	Hybrids *sampling time	12	60.36^**^	*p* < 0.01
Phosphorus	Hybrids	4	188.27^**^	*p* < 0.01
	sampling time	3	274.71^**^	*p* < 0.01
	Hybrids *sampling time	12	10.58^**^	*p* < 0.01
Lutein	Hybrids	4	10908.67^**^	*p* < 0.01
	sampling time	3	11426.57^**^	*p* < 0.01
	Hybrids *sampling time	12	1341.29^**^	*p* < 0.01
Zeaxanthin	Hybrids	4	3886.71^**^	*p* < 0.01
	sampling time	3	51308.30^**^	*p* < 0.01
	Hybrids *sampling time	12	482.53^**^	*p* < 0.01
β-criptoxanthin	Hybrids	4	608.78^**^	*p* < 0.01
	sampling time	3	2243.50^**^	*p* < 0.01
	Hybrids *sampling time	12	49.76^**^	*p* < 0.01
α-carotene	Hybrids	4	476.14^**^	*p* < 0.01
	sampling time	3	256.46^**^	*p* < 0.01
	Hybrids *sampling time	12	1.46^ns^	*p* ≥ 0.05
9Z-β-carotene	Hybrids	4	99.83^**^	*p* < 0.01
	sampling time	3	261.89^**^	*p* < 0.01
	Hybrids *sampling time	12	1.61^ns^	*p* ≥ 0.05
β-carotene	Hybrids	4	1064.71^**^	*p* < 0.01
	sampling time	3	881.33^**^	*p* < 0.01
	Hybrids *sampling time	12	153.85^**^	*p* < 0.01

### Correlation analysis

Correlation analysis revealed that dry matter was significantly and negatively correlated with fructose, glucose, calcium, iron, potassium, magnesium, zinc, phosphorus, α-carotene, 9Z-β-carotene, and β-carotene. Conversely, dry matter exhibited significant positive correlations with sucrose, lutein, zeaxanthin, and β-cryptoxanthin.

Fructose showed significant positive correlations with glucose, calcium, potassium, magnesium, zinc, phosphorus, α-carotene, 9Z-β-carotene, and β-carotene, while also presenting significant negative correlations with sucrose, zeaxanthin, and β-cryptoxanthin. Glucose was significantly and positively correlated with calcium, potassium, magnesium, zinc, phosphorus, α-carotene, 9Z-β-carotene, and β-carotene, and significantly negatively correlated with sucrose, zeaxanthin, and β-cryptoxanthin. Sucrose exhibited significant positive correlations with lutein, zeaxanthin and β-cryptoxanthin, but showed significant negative correlations with calcium, potassium, magnesium, zinc, phosphorus, α-carotene, 9Z-β-carotene, and β-carotene.

Calcium had significant positive correlations with potassium, magnesium, zinc, phosphorus, α-carotene, and 9Z-β-carotene, and β-carotene while showing significant negative correlations with lutein, zeaxanthin, and β-cryptoxanthin. Iron was significantly and positively correlated with potassium, magnesium, zinc, phosphorus, α-carotene, and 9Z-β-carotene, and β-carotene. Potassium exhibited significant positive correlations with magnesium, zinc, phosphorus, α-carotene, 9Z-β-carotene, and β-carotene. Moreover, potassium, magnesium, zinc, and phosphorus showed significant negative correlations with zeaxanthin and β-cryptoxanthin.

Magnesium was positively and significantly correlated with zinc, phosphorus, α-carotene, 9Z-β-carotene, and β-carotene. Zinc had significant positive correlations with phosphorus, α-carotene, 9Z-β-carotene, and β-carotene. Phosphorus was significantly and positively correlated with α-carotene, 9Z-β-carotene, and β-carotene. Lutein showed significant positive correlations with zeaxanthin and β-cryptoxanthin.

**Fig. 2:**
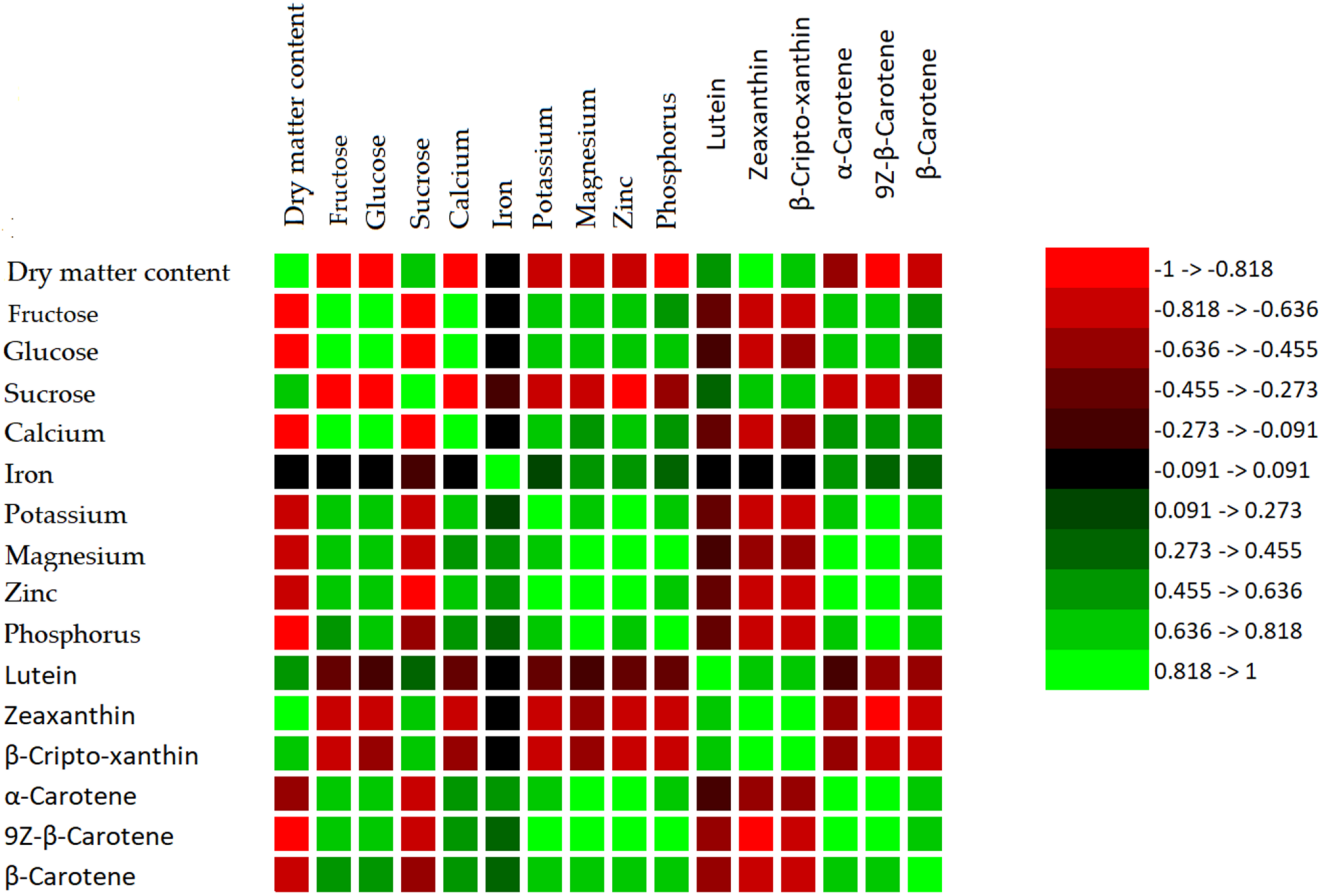
Heatmap of Correlation Analysis Among Biochemical and Nutritional Parameters in Crop Samples. This figure displays a correlation heatmap among various parameters, including sugars (fructose, glucose, sucrose), minerals (calcium, iron, potassium, magnesium, zinc, phosphorus), and carotenoids (lutein, zeaxanthin, β-cripto-xanthin, α-carotene, 9Z-β-carotene, β-carotene), along with dry matter content. The color scale represents the strength and direction of correlations ranging from strong negative (dark red) to strong positive (bright green).

Zeaxanthin was positively correlated with β-cryptoxanthin and negatively correlated with α-carotene, 9Z-β-carotene, and β-carotene. β-Cryptoxanthin exhibited significant negative correlations with α-carotene, 9Z-β-carotene and β-carotene. α-Carotene was positively correlated with 9Z-β-carotene and β-carotene. Finally, 9Z-β-carotene showed a significant positive correlation with β-carotene (Fig. 2). Correlation analysis revealed distinct groupings among traits, highlighting metabolic trade-offs. Dry matter content was negatively correlated with most minerals and carotenoids but positively associated with sucrose, lutein, zeaxanthin, and β-cryptoxanthin. Sugars (glucose and fructose) were positively correlated with each other, minerals, and major carotenoids, but negatively with sucrose and some xanthophylls. Minerals clustered with carotenoids, particularly β-carotene, α-carotene, and 9Z-β-carotene, while xanthophylls like zeaxanthin and β-cryptoxanthin showed positive associations with lutein and sucrose but negative correlations with minerals and carotenes. These relationships were summarized in a heatmap (Fig. 2).

### PCA analysis

**Fig. 3:**
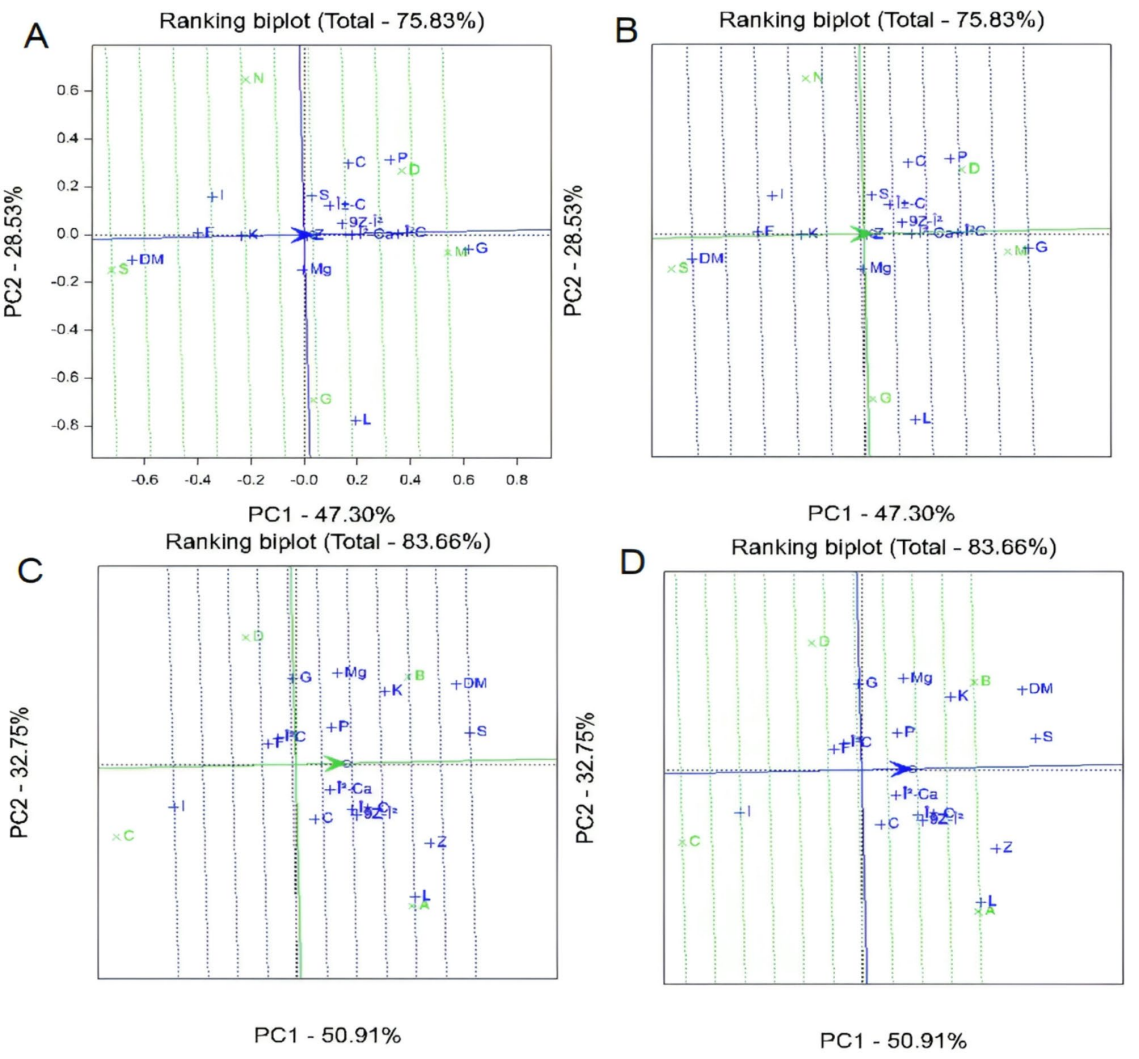
Principal Component Analysis (PCA) biplots illustrating the interaction between biochemical parameters, sampling times, and sweet corn hybrids. Variables include dry matter content (DM), fructose (F), glucose (G), sucrose (S), calcium (Ca), iron (Fe), potassium (K), magnesium (Mg), zinc (Zn), phosphorus (P), lutein (L), zeaxanthin (Z), β-cryptoxanthin (βC), α-carotene (αC), 9Z-β-carotene (9Z-βC), and β-carotene (β-Ca). Panels represent different sampling dates: (**A**) 19 July, (**B**) 26 July, (**C**) 2 August, and (**D**) 9 August. Hybrids are indicated by D, M, G, S, and N.

The PCA of biochemical parameters across sweet maize hybrids provided a comprehensive overview of genotype performance and trait variability. The first two principal components explained 75.82% of the total variance (PC1: 47.30%; PC2: 28.52%), demonstrating the robustness of the model in summarizing complex trait interactions. PC1 primarily reflected nutrient density, with strong positive loadings for glucose, β-carotene, phosphorus, and potassium, and negative loadings for dry matter and sucrose. PC2 captured variation in xanthophylls (lutein, zeaxanthin) and iron. The biplot analysis revealed that M hybrids clustered positively on both PCs, indicating superior biochemical accumulation and overall nutritional performance (Fig. 3A). D and G hybrids exhibited moderate performance with distinct xanthophyll and sucrose profiles, while the S hybrid consistently clustered negatively, reflecting lower values across biochemical traits. Glucose emerged as the most influential parameter driving hybrid differentiation, whereas dry matter contributed the least to overall variation (Fig. 3B). Ranking analysis highlighted potassium, lutein, zeaxanthin, sucrose, and dry matter as the highest-performing traits across hybrids, underscoring their importance as nutritional targets in sweet maize breeding. Conversely, iron consistently ranked lowest, signaling a potential focus area for improvement (Fig. 3C). Temporal analysis via the ranking biplot of sampling times indicated that early harvests on July 19 and July 26 yielded the most favorable biochemical profiles, suggesting these dates as optimal for maximizing nutritional quality. The August 2 harvest displayed the weakest profile, implying that delayed harvesting beyond late July may reduce key nutrient concentrations (Fig. 3D). Collectively, these PCA and biplot analyses underscore the critical influence of hybrid selection and harvest timing on sweet maize nutritional quality, offering valuable guidance for breeding and crop management strategies aimed at enhancing nutrient-rich maize varieties with high market and health value.

**Fig. 4:**
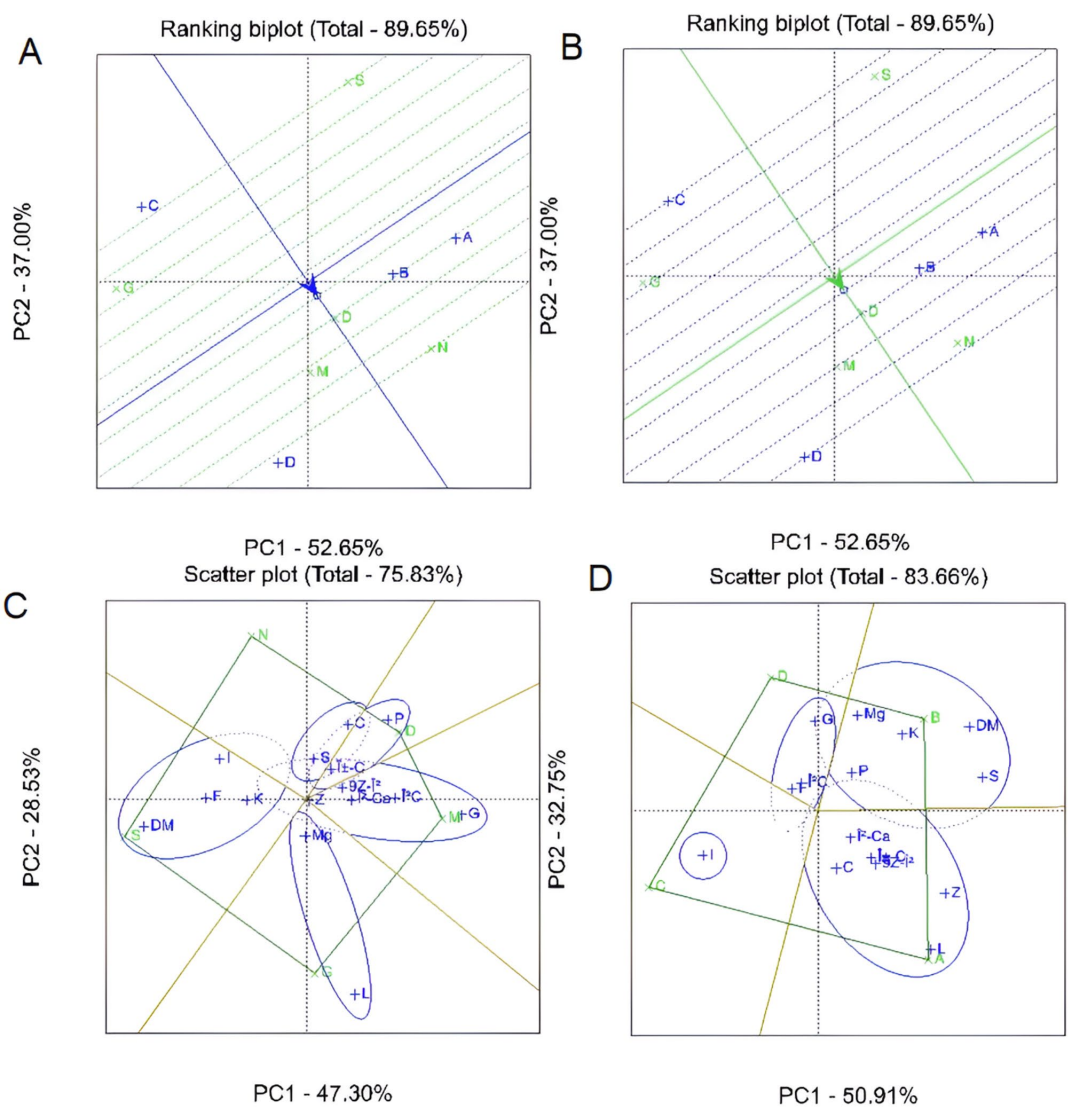
Principal Component Analysis (PCA) biplots and scatter plots illustrating the interaction among biochemical parameters, sampling times, and sweet corn hybrids. Traits analyzed include dry matter content (DM), fructose (F), glucose (G), sucrose (S), calcium (Ca), iron (Fe), potassium (K), magnesium (Mg), zinc (Zn), phosphorus (P), lutein (L), zeaxanthin (Z), β-cryptoxanthin (βC), α-carotene (αC), 9Z-β-carotene (9Z-βC), and β-carotene (β-Ca). Panels represent different sampling dates: (**A**) 19 July, (**B**) 26 July, (**C**) 2 August, and (**D**) 9 August. Hybrids are indicated by D, M, G, S, and N.

The biplot analysis examining sampling times in relation to genotypes revealed significant variations in hybrid performance across harvest dates. Among the evaluated hybrids, the N hybrid consistently demonstrated superior performance across all sampling times, indicating strong adaptability and stable expression of key biochemical traits (Fig. 4A). In contrast, the S hybrid showed the weakest performance throughout the study, suggesting limited adaptability under the tested environmental and management conditions. The genotype × sampling time biplot further highlighted that the harvest on August 9 produced the best genotype-specific performance, underscoring this date as optimal for maximizing the biochemical potential of the evaluated hybrids (Fig. 4B). Notably, glucose, phosphorus, and lutein displayed wide variation among hybrids, as indicated by scatter plot analyses, revealing significant genotype-dependent differences in the accumulation of these compounds despite controlled experimental conditions (Fig. 4C). Other biochemical traits exhibited more stable trends across hybrids, suggesting consistent expression independent of harvest timing. Individual biochemical trait biplots identified potassium, magnesium, glucose, phosphorus, calcium, 9Z-β-carotene, and iron as consistently high-performing across all sampling dates (Fig. 4D). These nutrients and bioactive compounds appear less sensitive to temporal variation, making them reliable targets for breeding programs focused on nutritional enhancement. Overall, these findings emphasize the complex interaction between genotype and harvest timing in shaping sweet maize’s nutritional and biochemical profiles. The stable performance of key hybrids such as N and M, contrasted with the limited adaptability of S, provides valuable guidance for cultivar selection and optimal harvest scheduling to improve crop value. Additionally, the observed variation in carotenoids—particularly β-carotene, α-carotene, and 9Z-β-carotene—has important nutritional implications. High β-carotene levels (notably in M hybrids at early harvest) support vitamin A biosynthesis and eye health. Low sucrose content may benefit diabetic diets or low-sugar processing, whereas higher sucrose (e.g., in D hybrid) enhances sweetness and consumer appeal. Elevated potassium, calcium, and magnesium concentrations contribute to cardiovascular and bone health, underscoring the value of nutrient-dense hybrids like M and N.Benchmark nutritional thresholds such as β-carotene > 2 µg/g FW, potassium > 350 mg/100 g, and magnesium > 30 mg/100 g delineate desirable profiles. Moreover, β-cryptoxanthin—a provitamin A carotenoid associated with reduced cancer risk and oxidative stress—highlights the importance of hybrids rich in xanthophylls for health-promoting maize varieties.

## Discussion

This study demonstrated that both harvest timing and hybrid genotype significantly influence the biochemical composition of sweet maize, corroborating previous findings that variety, environmental conditions, and maturity stages critically affect maize quality^14,15^. Variance analysis revealed distinct nutrient‑accumulation patterns among hybrids and sampling times, underscoring the dynamic nature of sweet‑maize biochemical traits. The M hybrid consistently showed superior accumulation of key nutrients such as β‑carotene, α‑carotene, phosphorus, and essential minerals like zinc and magnesium. This aligns with PCA results that ranked M highest in overall biochemical performance, indicating strong potential for breeding programs focused on nutritional enhancement^16,17^. Biological and practical relevance. From a nutritional‑security standpoint, the 1.5‑ to 2‑fold higher provitamin‑A carotenoid concentrations observed in M (and, to a lesser extent, N) at the earliest harvest translate into an estimated 25–35% greater contribution toward daily vitamin‑A requirements for children and pregnant women—populations most vulnerable to micronutrient deficiencies. At the same time, elevated zinc and magnesium levels improve the ionome balance critical for enzymatic activity and immune function. For producers, these biochemical advantages can be leveraged to market “nutrient‑dense” sweet‑corn products that command premium prices in fresh‑produce segments and functional‑food formulations, thereby expanding revenue streams beyond bulk sweet‑corn markets. Conversely, processors targeting maximal sugar yield for canning or syrup manufacture may opt for later harvests despite the concomitant decline in carotenoids, illustrating how the results provide a decision framework for aligning harvest schedules with end‑use quality goals. Conversely, the S hybrid, despite its reported higher sucrose content and grain yield in other studies^18^, exhibited the lowest overall biochemical quality in this study, highlighting that elevated sugar levels do not necessarily translate to improved nutritional profiles. (Yield data were not collected here, so no explicit claims regarding yield performance are made.) Instead, observed variations in key compounds—such as β‑carotene, glucose, phosphorus, and potassium—highlight the physiological potential of certain hybrids, particularly M and N, for producing nutrient‑dense crops. Harvest timing played a crucial role: the earliest harvest (19 July, sampling time A) yielded the highest concentrations of vital nutrients, including β‑carotene and essential minerals, supporting earlier research emphasizing optimal harvest timing to maximize nutritional content^19,20^. Later harvests showed increased dry matter and sucrose but reduced carotenoid concentrations, consistent with kernel‑maturation processes that dilute certain bioactive compounds^21,22^. These temporal shifts illustrate the trade‑offs producers face between yield, processing quality, and nutrient density; by quantifying these trade‑offs, the study provides actionable guidance for growers, breeders, and food companies seeking to tailor sweet‑corn products to specific market niches (fresh‑market, processing, or nutraceutical). Strong positive correlations between glucose and various minerals and carotenoids suggest linked metabolic regulation, while negative correlations between dry matter and sugars reflect biochemical shifts during kernel desiccation and maturation. Glucose emerged as a critical biochemical marker differentiating hybrids, consistent with previous biochemical and genomic studies^23^. The N hybrid demonstrated remarkable stability across sampling times, marking it as a promising candidate for environments with variable harvest schedules and for breeding programs aiming to deliver consistent nutritional quality. In contrast, the S hybrid’s poor adaptability limits its broader cultivation potential despite its high sucrose content. Although yield and fertilizer responses were not evaluated here, existing literature suggests hybrids with superior biochemical profiles like M and N also perform well agronomically under optimized nutrient management^15,18^. Integrating yield data in future trials will be essential to confirm whether the nutritional superiority observed here translates into commercially viable productivity. The observed biochemical shift during kernel maturation—declining glucose coupled with rising sucrose concentrations—reflects carbohydrate‑metabolism dynamics in which glucose is converted into sucrose for transport and storage, while increasing dry matter reflects starch accumulation and kernel desiccation^21,22^. This creates a trade‑off for producers: delayed harvest may improve yield and sweetness, valuable for processing and consumer taste, but reduces carotenoid and mineral content critical for nutritional value. These findings emphasize the need to optimize harvest timing to balance yield, sensory quality, and nutrient density, which is particularly relevant for value‑added markets promoting biofortified or “eye‑health” corn products. Genotypic variation further modulates these effects, with M and N hybrids showing superior accumulation of carotenoids, phosphorus, and essential minerals, whereas S, despite its higher sucrose, exhibits lower nutritional quality. These differences likely reflect genetic regulation of metabolic pathways controlling nutrient biosynthesis and uptake^16,23^. To enhance the interpretation of results, we integrated findings from ANOVA, correlation, and PCA to reveal a consistent pattern in hybrid performance and trait interactions. ANOVA identified significant effects of both genotype and harvest timing on nearly all biochemical traits, with LSD grouping confirming hybrid M’s superior accumulation of β-carotene, glucose, and multiple essential minerals. These results were corroborated by the PCA, where M clustered positively on PC1, a component heavily loaded with glucose, β-carotene, phosphorus, and potassium—traits also shown by ANOVA to vary significantly among hybrids. Similarly, hybrid S, identified by ANOVA as having high levels of xanthophylls but lower mineral content, clustered negatively on PC1 and positively on PC2, where xanthophylls and iron contributed most. Correlation analysis further supported these patterns, revealing strong positive associations between glucose and nutrient minerals like phosphorus and zinc—key contributors to PC1—highlighting shared metabolic regulation. The negative correlation between dry matter and sugars also mirrored PC1’s structure, where dry matter loaded negatively and glucose positively. These converging patterns across statistical approaches demonstrate that PCA not only confirms ANOVA-based trait differentiation but also provides a multivariate framework to interpret complex trait relationships, strengthening the biological relevance of the observed genotype × harvest interactions. The N hybrid’s stability across sampling times suggests genetic resilience to environmental and developmental variation, making it an ideal candidate for breeding programs targeting nutritional consistency. Conversely, S hybrid’s limited adaptability despite sugar advantage highlights a trade‑off between yield traits and biochemical quality. Practical takeaway. For breeders, our data identify M and N as promising parents for pyramiding carotenoid‑ and mineral‑enhancing alleles without sacrificing sweetness. For growers and processors, the quantified nutrient‑versus‑sugar curves provide an evidence base for tailoring harvest windows to specific market demands—be it fresh, processing, or functional‑food sectors. Finally, consumers stand to benefit from sweet‑corn products that contribute meaningfully to daily micronutrient intake while retaining desirable flavor. Future research integrating yield and nutrient‑management data—and evaluating consumer acceptance—will strengthen understanding of these complex interactions and inform the development of nutritionally enhanced, agronomically viable hybrids.

## Conclusion

The M hybrid emerged as the best-performing genotype regarding nutritional quality, while mid to late July harvests (specifically July 19 and July 26) were optimal for maximizing biochemical content. These findings offer clear guidance for breeders and producers aiming to enhance sweet maize nutritional value, and highlight the critical role of coordinated genotype selection and harvest timing in improving crop quality. The superior performance of the M hybrid across multiple traits particularly β-carotene, phosphorus, potassium, and glucose demonstrates its strong potential as a candidate for biofortification and health-oriented breeding programs. Additionally, the early harvest windows coincide with peak accumulation of essential sugars and provitamin A carotenoids, ensuring optimal nutritional content before kernel maturation dilutes key compounds. By identifying both stable high-performing genotypes and the most advantageous harvest windows, this study provides practical recommendations that can be directly applied in commercial cultivation, seed selection, and varietal development. Furthermore, the integration of PCA, correlation analysis, and ANOVA offers a robust framework for understanding genotype × environment interactions, which can inform precision agriculture approaches and targeted management strategies. Overall, the strategic alignment of hybrid choice and timely harvest enables producers to enhance not only the market appeal of sweet maize but also its contribution to public health and sustainable nutrition.

## Electronic supplementary material

Below is the link to the electronic supplementary material.

Supplementary Material 1.

## Data Availability

The datasets used and/or analysed during the current study available from the corresponding author on reasonable request.
