# Supplementary material for: Effect of harvest time on sugar content and carotenoid composition in different sweet maize hybrids
Source: Sci Rep. 2025 Jul 16;15:25758. doi: 10.1038/s41598-025-11680-w (PMC12267483; doi:10.1038/s41598-025-11680-w)
Supplement: Supplementary file 1 — Supplementary Material 1. [file 41598_2025_11680_MOESM1_ESM.docx]

Grouping genotype based on LSD

|  | Name of hybrid | N | Mean | Grouping | | | |  |
| --- | --- | --- | --- | --- | --- | --- | --- | --- |
| β-Carotene | M | 16 | 0.578022 | A |  |  |  |  |
|  | G | 16 | 0.381892 |  | B |  |  |  |
|  | N | 16 | 0.376238 |  | B |  |  |  |
|  | D | 16 | 0.319418 |  |  | C |  |  |
|  | S | 16 | 0.254852 |  |  |  | D |  |
| 9Z-β-Carotene | M | 16 | 0.68125 | A |  |  |  |  |
|  | N | 16 | 0.47500 |  | B |  |  |  |
|  | D | 16 | 0.43125 |  | B | C |  |  |
|  | G | 16 | 0.38125 |  |  | C | D |  |
|  | S | 16 | 0.36250 |  |  |  | D |  |
| α-Carotene | M | 16 | 0.95625 | A |  |  |  |  |
|  | N | 16 | 0.57500 |  | B |  |  |  |
|  | S | 16 | 0.51250 |  |  | C |  |  |
|  | D | 16 | 0.33750 |  |  |  | D |  |
|  | G | 16 | 0.26250 |  |  |  |  | E |
| β-Cripto-xanthin | S | 16 | 2.24489 | A |  |  |  |  |
|  | D | 16 | 1.46754 |  | B |  |  |  |
|  | G | 16 | 1.26991 |  |  | C |  |  |
|  | M | 16 | 1.17374 |  |  |  | D |  |
|  | N | 16 | 1.09543 |  |  |  |  | E |
| Zeaxanthin | S | 16 | 23.0699 | A |  |  |  |  |
|  | D | 16 | 17.8833 |  | B |  |  |  |
|  | N | 16 | 14.0181 |  |  | C |  |  |
|  | G | 16 | 13.1412 |  |  |  | D |  |
|  | M | 16 | 8.9547 |  |  |  |  | E |
| Lutein | S | 16 | 8.72369 | A |  |  |  |  |
|  | N | 16 | 3.44884 |  | B |  |  |  |
|  | D | 16 | 3.24080 |  |  | C |  |  |
|  | M | 16 | 2.06805 |  |  |  | D |  |
|  | G | 16 | 1.71402 |  |  |  |  | E |
| Phosphorus | M | 16 | 3639.43 | A |  |  |  |  |
|  | D | 16 | 3283.63 |  | B |  |  |  |
|  | G | 16 | 3218.85 |  |  | C |  |  |
|  | S | 16 | 3188.01 |  |  | C |  |  |
|  | N | 16 | 3104.55 |  |  |  | D |  |
| Zinc | M | 16 | 36.7317 | A |  |  |  |  |
|  | N | 16 | 30.3882 |  | B |  |  |  |
|  | S | 16 | 27.4353 |  |  | C |  |  |
|  | G | 16 | 26.4143 |  |  |  | D |  |
|  | D | 16 | 25.5652 |  |  |  |  | E |
| Magnesium | M | 16 | 1887.46 | A |  |  |  |  |
|  | S | 16 | 1298.11 |  | B |  |  |  |
|  | N | 16 | 1293.68 |  | B |  |  |  |
|  | G | 16 | 1193.35 |  |  | C |  |  |
|  | D | 16 | 1138.37 |  |  |  | D |  |
| Potassium | M | 16 | 13444.1 | A |  |  |  |  |
|  | N | 16 | 11396.4 |  | B |  |  |  |
|  | D | 16 | 10931.5 |  |  | C |  |  |
|  | S | 16 | 9955.8 |  |  |  | D |  |
|  | G | 16 | 9337.7 |  |  |  |  | E |
| Iron | M | 16 | 22.3267 | A |  |  |  |  |
|  | S | 16 | 17.4791 |  | B |  |  |  |
|  | N | 16 | 17.4744 |  | B |  |  |  |
|  | D | 16 | 16.7783 |  |  | C |  |  |
|  | G | 16 | 14.8373 |  |  |  | D |  |
| Calcium | M | 16 | 219.926 | A |  |  |  |  |
|  | G | 16 | 167.892 |  | B |  |  |  |
|  | S | 16 | 158.457 |  |  | C |  |  |
|  | N | 16 | 140.569 |  |  |  | D |  |
|  | D | 16 | 107.691 |  |  |  |  | E |
| Sucrose | D | 16 | 29.0459 | A |  |  |  |  |
|  | S | 16 | 26.8664 |  | B |  |  |  |
|  | G | 16 | 24.3443 |  |  | C |  |  |
|  | M | 16 | 21.9484 |  |  |  | D |  |
|  | N | 16 | 21.3482 |  |  |  | D |  |
| Glucose | M | 16 | 14.1375 | A |  |  |  |  |
|  | S | 16 | 13.2938 |  | B |  |  |  |
|  | G | 16 | 12.4375 |  |  | C |  |  |
|  | N | 16 | 12.4375 |  |  | C |  |  |
|  | D | 16 | 8.5625 |  |  |  | D |  |
| Fructose | N | 16 | 11.3125 | A |  |  |  |  |
|  | M | 16 | 11.2250 | A |  |  |  |  |
|  | S | 16 | 10.0563 |  | B |  |  |  |
|  | G | 16 | 9.8750 |  | B |  |  |  |
|  | D | 16 | 6.6875 |  |  | C |  |  |
| Dry matter | N | 16 | 26.2196 | A |  |  |  |  |
|  | S | 16 | 25.0907 |  | B |  |  |  |
|  | D | 16 | 24.1612 |  |  | C |  |  |
|  | G | 16 | 23.0719 |  |  |  | D |  |
|  | M | 16 | 19.3426 |  |  |  |  | E |

Compare with LSD grouping showed that A sampling time had maximum performance on β-Carotene, 9Z-β-Carotene, α-Carotene, Phosphorus, Zinc, Magnesium, Potassium, Calcium, Glucose, Fructose; B sampling times had maximum performance on Iron, C sampling times had maximum performance on Lutein, and D sampling times had maximum performance on β-Cripto-xanthin, Zeaxanthin, Sucrose, and Dry Matter.

Grouping sampling time based on LSD

|  | Sampling time | N | Mean | Grouping | | | |
| --- | --- | --- | --- | --- | --- | --- | --- |
| β-Carotene | 19 July | 20 | 0.459399 | A |  |  |  |
|  | 26 July | 20 | 0.416211 |  | B |  |  |
|  | 02 Aug | 20 | 0.327538 |  |  | C |  |
|  | 09Aug | 20 | 0.276523 |  |  |  | D |
| 9Z-β-Carotene | 19 July | 20 | 0.675 | A |  |  |  |
|  | 26 July | 20 | 0.545 |  | B |  |  |
|  | 02 Aug | 20 | 0.400 |  |  | C |  |
|  | 09Aug | 20 | 0.245 |  |  |  | D |
| α-Carotene | 19 July | 20 | 0.735 | A |  |  |  |
|  | 26 July | 20 | 0.600 |  | B |  |  |
|  | 02 Aug | 20 | 0.455 |  |  | C |  |
|  | 09Aug | 20 | 0.325 |  |  |  | D |
| β-Cripto-xanthin | 09Aug | 20 | 2.23392 | A |  |  |  |
|  | 02 Aug | 20 | 1.84440 |  | B |  |  |
|  | 26 July | 20 | 1.29742 |  |  | C |  |
|  | 19 July | 20 | 0.61082 |  |  |  | D |
| Zeaxanthin | 09Aug | 20 | 32.7214 | A |  |  |  |
|  | 02 Aug | 20 | 28.0279 |  | B |  |  |
|  | 26 July | 20 | 16.1865 |  |  | C |  |
|  | 19 July | 20 | 1.8369 |  |  |  | D |
| Lutein | 02 Aug | 20 | 5.78465 | A |  |  |  |
|  | 09Aug | 20 | 4.23565 |  | B |  |  |
|  | 26 July | 20 | 4.00118 |  |  | C |  |
|  | 19 July | 20 | 1.04665 |  |  |  | D |
| Phosphorus | 19 July | 20 | 3542.09 | A |  |  |  |
|  | 26 July | 20 | 3356.14 |  | B |  |  |
|  | 02 Aug | 20 | 3203.02 |  |  | C |  |
|  | 09Aug | 20 | 3047.63 |  |  |  | D |
| Zinc | 19 July | 20 | 35.8989 | A |  |  |  |
|  | 26 July | 20 | 30.9266 |  | B |  |  |
|  | 02 Aug | 20 | 26.1402 |  |  | C |  |
|  | 09Aug | 20 | 24.4031 |  |  |  | D |
| Magnesium | 19 July | 20 | 1630.11 | A |  |  |  |
|  | 26 July | 20 | 1484.36 |  | B |  |  |
|  | 02 Aug | 20 | 1215.73 |  |  | C |  |
|  | 09Aug | 20 | 1103.03 |  |  |  | D |
| Potassium | 19 July | 20 | 14186.3 | A |  |  |  |
|  | 26 July | 20 | 12166.5 |  | B |  |  |
|  | 02 Aug | 20 | 10173.0 |  |  | C |  |
|  | 09Aug | 20 | 8574.1 |  |  |  | D |
| Iron | 26 July | 20 | 18.5073 | A |  |  |  |
|  | 02 Aug | 20 | 18.1248 | A |  |  |  |
|  | 09Aug | 20 | 17.3073 |  | B |  |  |
|  | 19 July | 20 | 16.5971 |  |  | C |  |
| Calcium | 19 July | 20 | 576.829 | A |  |  |  |
|  | 26 July | 20 | 124.866 |  | B |  |  |
|  | 02 Aug | 20 | 95.454 |  |  | C |  |
|  | 09Aug | 20 | 83.282 |  |  |  | D |
| Sucrose | 09Aug | 20 | 29.7597 | A |  |  |  |
|  | 02 Aug | 20 | 26.9822 |  | B |  |  |
|  | 26 July | 20 | 24.5598 |  |  | C |  |
|  | 19 July | 20 | 18.0128 |  |  |  | D |
| Glucose | 19 July | 20 | 18.450 | A |  |  |  |
|  | 26 July | 20 | 12.060 |  | B |  |  |
|  | 02 Aug | 20 | 10.160 |  |  | C |  |
|  | 09Aug | 20 | 8.025 |  |  |  | D |
| Fructose | 19 July | 20 | 15.115 | A |  |  |  |
|  | 26 July | 20 | 10.555 |  | B |  |  |
|  | 02 Aug | 20 | 7.955 |  |  | C |  |
|  | 09Aug | 20 | 5.700 |  |  |  | D |
| Dry Matter | 09Aug | 20 | 30.8618 | A |  |  |  |
|  | 02 Aug | 20 | 28.4509 |  | B |  |  |
|  | 26 July | 20 | 23.2732 |  |  | C |  |
|  | 19 July | 20 | 13.5685 |  |  |  | D |
